# Effect of Gamma Irradiation on the Osteoinductivity of Demineralized Dentin Matrix for Allografts: A Preliminary Study

**DOI:** 10.3390/jfb13010014

**Published:** 2022-01-31

**Authors:** Jeong-Kui Ku, Il-hyung Kim, In-Woong Um, Bo-Hyun Kim, Pil-Young Yun

**Affiliations:** 1 Department of Oral and Maxillofacial Surgery, Gangnam Severance Hospital, Yonsei University College of Dentistry, Seoul 06273, Korea; kujk123@gmail.com; 2 Department of Oral and Maxillofacial Surgery, Armed Forces Capital Hospital, Armed Forces Medical Command, Seongnam-si 13574, Korea; haonflower@gmail.com; 3 R&D Institute, Korea Tooth Bank, Seoul 06101, Korea; kbh8708@naver.com; 4 Department of Oral and Maxillofacial Surgery, Section of Dentistry, Seoul National University Bundang Hospital, Bundang-gu, Seongnam-si 13620, Korea

**Keywords:** bone graft substitutes, demineralized dentin matrix, osteoinductivity, gamma irradiation

## Abstract

Demineralized dentin matrix (DDM) treated with gamma irradiation (GR) has shown promising results as an allograft without any adverse effects in in vivo and clinical studies. The purpose of this study was to evaluate the effects of 15 and 25 kGy GR on the osteoinductive properties of DDM at extra-skeletal sites. As a control group, non-irradiated DDM powder was implanted into the right subcutaneous tissues of the dorsal thigh muscles of 20 nude mice. DDM powder irradiated with 15 and 25 kGy was implanted into the left side. After two and four weeks, the bone mineral density (BMD) was measured with dual-energy X-ray absorptiometry. After confirming osteoblast- and osteoclast-specific activities by alkaline phosphatase (ALP) and tartrate-resistant acid phosphatase (TRAP) staining, a histological analysis was performed to measure the new bone formation and the number of osteoblasts and osteoclast-like cells on the surface of the DDMs. Histomorphometry was used to calculate the new bone formation area on the surface of the DDM particles (DDMs). The BMD in all the groups increased from two and four weeks without statistically significant differences. The osteoblasts were dominantly activated on DDM without GR, and DDM treated with 25 kGy compared to DDM treated with 15 kGy. Among the groups, new bone formation was identified in all the groups at each time point. In conclusion, GR at doses of 15 and 25 kGy does not affect the osteoinductive properties of DDM powder.

## 1. Introduction

Demineralized dentin matrix (DDM), which is obtained by removing inorganic salts with minimal leaching without denaturing the organic components of the matrix, is defined as an acid-insoluble, highly cross-linked type I collagen with matrix-binding proteins such as bone morphogenetic proteins (BMPs) [1]. Bang and Urist first reported the bone induction of allogenic, isogeneic, and xenogeneic DDM in rabbit and rat abdominal muscles [2]. Osteoinductivity of DDM has been revealed in several in vitro, in vivo, and clinical studies [3,4]. Autogenous DDM (auto-DDM) has shown promising clinical and histological results that are comparable to autogenous bone grafts [5]. However, the need for an allogeneic DDM (allo-DDM) has emerged to overcome the disadvantages of auto-DDM, such as an insufficient quantity that is dependent on the extracted teeth and the delayed time from extraction to grafting [6].

Regarding allogeneic bone substitutes, demineralized bone matrix (DBM) has been widely used in patients with skeletal defects and periodontal diseases [7]. Both DDM and DBM are predominantly composed of type I collagen (95%), and BMPs bind to type I collagen that belongs to the dentin and bone matrix, even after complete demineralization [8]. The Allo-DDM process generally stems from DBM, which was largely developed by Urist in 1967. Allo-DDM is a refined allograft that has greater biocompatibility, and it reduces the probability of transmitting some disease [9,10]. However, it is well known that biological osteoinductivity is decreased or diminished by the demineralization process, which includes chemicals, heating, and irradiation [11,12,13].

A variety of allograft processing procedures have aimed to assure virus inactivation using detergents, freeze-drying, chemical sterilization, and gamma irradiation (GR). These have been evaluated and result in an exponential reduction in the potential for graft contamination, disease transfer, or both [14,15,16]. Among these procedures, GR is a simple, cost-effective, and very favorable sterilization technique for bone allografts that is known to inactivate all types of viruses that are enveloped and non-enveloped [17,18,19]. Although viruses show maximum resistance against irradiation, several studies have shown that the hepatitis C virus (HCV) and human immunodeficiency virus (HIV) are completely vulnerable to high doses of radiation [20,21]. In 2020, the effect of gamma irradiation with 15 and 25 kGy was a significant reduction of the HBV DNA levels in the fresh dentin matrix that was harvested from chronically infected patients [22].

Although GR provides additional assurance of bone allograft safety from viral transmission, the major disadvantage of GR sterilization is its hazardous effects on the osteoinductivity of DBM [13,14]. Urist and Hernandez reported that gamma irradiation with a 40 kGy dose leads to diminished osteoinduction implant properties of demineralized bone powder [23]. Dziedzic-Goclawska et al. reported that 35–50 kGy gamma irradiation at room temperature could diminish the osteoinductive properties of the demineralized bone powder completely [24]. On the other hand, GR at 30 kGy was less damaging to osteoinductivity [10,25], which is representative of its effect from BMPs [26]. This was considered to be acceptable because most of the clinical applications involved intraosseous defects where some osteoconduction can be expected to contribute to healing [14]. According to the European Association of Tissue Banks (EATB) and the International Atomic Energy Agency (IAEA) guidelines, the permissible dosage for GR is 25 kGy, and the American Association of Tissue Banks (AATB) suggests a dose of 15 kGy, which has the potential to reduce the deleterious effects of GR on tissue properties that occur between 15 and 25 kGy [27,28,29,30].

Concerning DDM, because dentin matrix is different from bone matrix since there are no cells and vessels, DDM seems to be safer than DMB in terms of transmissible viral inactivation with similar osteoinductive functions [3,8]. However, the validation of viral safety seems to be mandatory for the allogeneic applications of DDM in dentistry. In addition to the processing procedures of DDM that could inactivate HBV on freshly infected dentin [31], Ku et al. recently reported GR treatment of fresh dentin from hepatitis B virus (HBV)-infected patients at dosages of 15 and 25 kGy. The authors suggested that GR treatment at 15 and 25 kGy is effective in the elimination or inactivation of HBV [22]. However, the effect of GR on the osteoinductivity of DDM has not been reported yet.

Based on these previous studies of DBM, which have similar components and functions to DDM, the aim of this study is to investigate the effects of GR sterilization at doses of 15 and 25 kGy on the osteoinductive properties of human DDM in the subcutaneous tissues of nude mice. We hypothesized that GR sterilization of DDM does not hinder the osteoinductivity of DDM.

## 2. Materials and Methods

This study was approved by the Institutional Animal Care Committee of Seoul National University Bundang Hospital (Seongman, Korea, SNUBH) (IACUC No. BA-2008-301-070-01). All the experiments were performed in compliance with the Guide for Experimental Animal Research of the Laboratory for Experimental Animal Research, Clinical Research Institute, SNUBH, South Korea, and were confirmed with the provisions of the Declaration of Helsinki.

### 2.1. Preparation of the Demineralized Dentin Matrix (DDM, Control Group) Powder

The DDM was purchased from Korea Tooth Bank (KTB, Seoul, Korea). It was fabricated according to the standardized procedures of the KTB and the Guidelines of Good Practice for Tooth Handling Institution from the Korea Ministry of Health and Welfare (KMHW) [32]. The conventional DDM without GR treatment that was prepared from human teeth was used as the control group. The other DDM groups that underwent GR at doses of 15 kGy (15DDM) and 25 kGy (25DDM) were the experimental group [33,34].

Briefly, the extracted human teeth were soaked in 70% ethyl alcohol, and they were cleaned by removing the soft tissues of the periodontium, pulp, and caries. After dividing the cleaned teeth into the crown and root, the root portion was collected and prepared as DDM. The crushed particles (300–800 μm) were soaked in distilled water and hydrogen dioxide solution and the remaining foreign substances were removed using an ultrasonic cleaner. The cleaned particles were dehydrated with ethyl alcohol and subjected to defatting using ethyl ether solution. The particles were then demineralized for 30 min in 0.6 N HCl. The demineralized particles were lyophilized, packed, and sterilized with ethylene oxide gas.

### 2.2. Radiation Sterilization (Experimental Groups)

GR was conducted using Greenpia Technology (Greenpia Technology Inc. R&D Center, Yeoju, Korea) by a gamma-ray (Co-60) survey facility instrument (JS-8900 MDS Nord International Co. Ltd., Ottawa, ON, Canada). The total absorbed dose of gamma radiation was obtained at room temperature at a dose rate of 1 kGy per unit of time. A dosimetry system (Harwell RED 4034 Dosimeters, Harwell Dosimeters LTD, Oxfordshire, UK) was used following the IAEA standards to verify the standardization of the absorbed doses, with a total absorbed dose error of less than 8%.

### 2.3. Experimental Animals

In this study, 20 male athymic nude mice (BALB/c, immune-deficient) were purchased from Orientbio© (aged 8 weeks, weighing 30–40 g, Seongnam, Korea) and were housed in four separate cages with a standard diet and water at the animal laboratory of the Seoul National University Bundang Hospital Animal Research Center. The room temperature was maintained at 22.24 °C, and it was synchronized for a light–dark cycle that was 12 h. All the protocols were carried out according to the Ethical Guidelines of the Animal Protection Association and were approved by the Animal Care and Ethics Committee.

### 2.4. Experimental Procedures

The mice were anesthetized by inhaling 2.2% isoflurane (Hana Pharm. Co., Seoul, Korea) and 2 L/min of oxygen. The site was disinfected with povidone-iodine. After infiltration of 1.8 mL of 2% lidocaine HCl (Huons^®^, Kyeongi-do, Korea) for local anesthesia, a 5 mm linear incision was made on both thighs of the mouse, and a subcutaneous dissection was performed to secure the space for the graft. To reduce the number of animals, 30 mg of DDM was grafted into the right subcutaneous pocket of the thigh, and 30 mg of 15 or 25DDM was grafted to the left. In this study, 20 nude mice were equally allocated to Group A (DDM and 15DDM; 5 mice for two and four weeks, respectively) and Group B (DDM and 25DDM; 5 mice for two and four weeks, respectively), respectively (Supplementary Figure S1).

After grafting, primary closure was achieved with 4-0 Vicryl (Ethicon, Inc., Somerville, NJ, USA). Following surgery, postoperative care was performed with gentamicin (JW Pharm. Co., Seoul, Korea, 10 mg/kg, SC) and ketorolac tromethamine (Bukwang Pharm. Co., Seoul, Korea, 5 mg/kg, SC) daily for three days to reduce the pain and prevent infection. The site was disinfected with a povidone-iodine solution for one week.

### 2.5. Bone Mineral Density Analysis

At two and four weeks postoperatively, the bone mineral density (BMD, g/cm^3^) was measured in five nude mice in each group using dual-energy X-ray absorptiometry (InAlyzer, Medikors, Seoul, Korea) immediately after the sacrifice through carbon dioxide euthanasia. The X-ray settings were as follows: energy of 50 kV and an intensity of 500 μA. According to the mass in the subcutaneous pocket, a 1 mm × 1 mm square area in the peripheral area was selected as the region of interest (ROI). From this, the parameter of the bone mineral density (BMD) was measured, and then it was compared between the groups.

### 2.6. Histological Analysis

**Figure 1:**
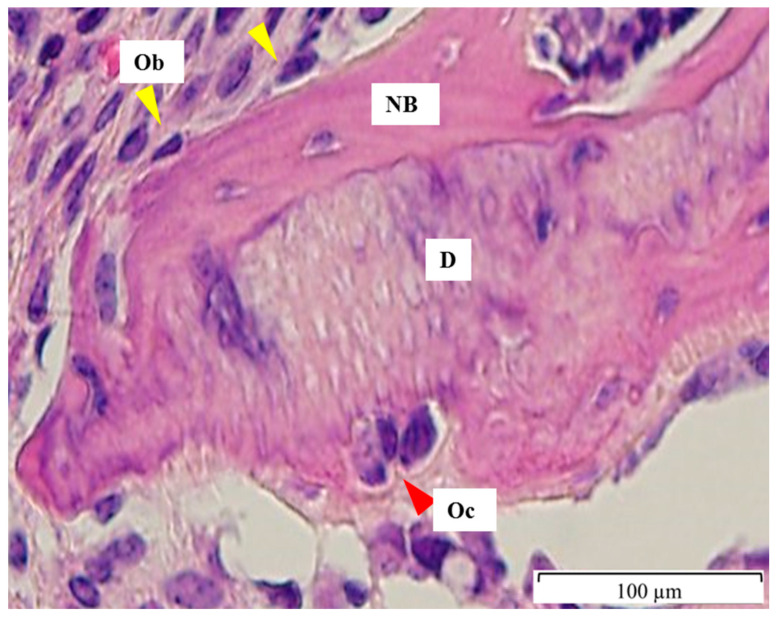
Numbers of osteoblasts and osteoclast-like cells on the surface of DDM at 4 weeks. Osteoblasts (Ob) are lined on the surface of newly formed osteoid (yellow arrowhead), while osteoclast-like cells (Oc) are on the ruffled border of DDM (D). D; DDM particle, NB; newly formed bone on DDM surface, Ob; Osteoblast, Oc; Osteoclast-like cells.

After taking the X-rays, the samples were carefully excised en bloc, including the thigh muscles, and they were decalcified using 10% ethylenediaminetetraacetic acid for 14 days at room temperature. The specimens were trimmed and embedded in paraffin. Serial sections of 4 μm were cut from each part and stained with the hematoxylin and eosin stain to evaluate the number of osteoblasts, osteoclast-like cells, and the amount of new bone formation on the surface of the DDM (Figure 1). In addition, alkaline phosphatase (ALP) and tartrate-resistant acid phosphatase (TRAP) staining were performed based on standard protocols (Cosmo Bio, Tokyo, Japan) to identify osteoblastic activity and osteoclast-like cells. The histological images were captured using a 100× objective lens with an Olympus BX50 microscope (Olympus DP72 camera, Olympus Corporation, Tokyo, Japan), and they were analyzed using the Olympus cellSens Standard 1.12 (Olympus Corporation) software program.

### 2.7. Number of Osteoblasts and Osteoclast-Like Cells on the Surface of the DDM

The number of osteoblasts and osteoclast-like cells surrounding the DDM particles was counted. First, a DDM particle was selected based on the available area surrounding the particle for the calculation. Then, the DDM particle boundary was marked using the closed polygon option under the measure tab of the cellSens software program. An equivalent area surrounding the DDM particle and new bone boundary was then taken by marking a boundary that was 25 μm from the dentin and the new bone boundary. The cells were counted within this area only (Supplementary Figure S2). All the evaluations were performed at a 20× magnification.

### 2.8. Quantitative Measurement of the New Bone Formation on the Surface of the DDM (%, µm^2^)

**Figure 2:**
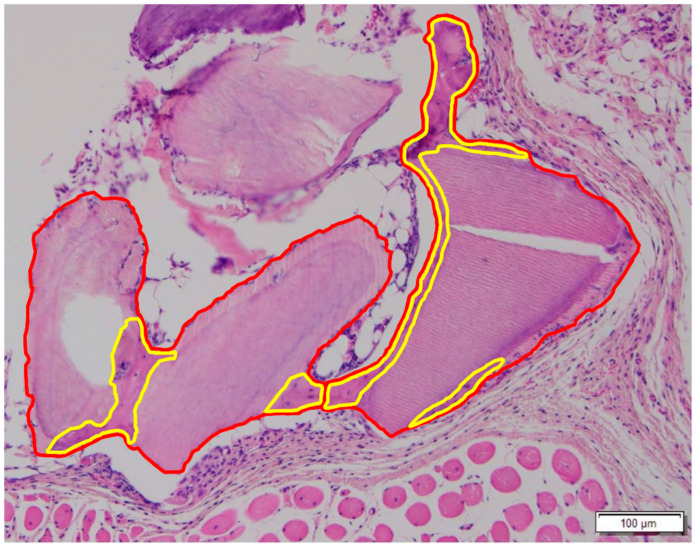
Measurement of new bone formation on the surface of the DDM. The area of the dentin particle and the newly formed bone is highlighted with the red line. The area of the new bone is highlighted with the yellow line, which shows embedded lining cells and distinguished cellularity from the acellular DDM.

The quantitative measurement of the new bone formation was performed in a similar way using the closed polygon option. From these values, the new bone (NB) % was calculated as the NB divided by the total bone (TB), which consists of NB and DDM particles (NB % = $\frac{NB\;area}{DDM+NB\;areas}$ × 100) (Figure 2).

### 2.9. Statistical Analysis

The normal distribution of all the data was confirmed by the Kolmogorov–Smirnov test and the data were expressed as the mean ± standard deviation. To compare the three grafts (DDM, 15DDM, and 25DDM), a statistical analysis was performed using single factor analysis of variance (one-way ANOVA with post-hoc analysis by the Bonferroni method) using the software program SPSS version 25.0 e (SPSS. Inc., Chicago, IL, USA). The significance was set at a level of 0.05.

## 3. Results

### 3.1. Representative Histological Images of DDM, 15DDM, and 25DDM

**Figure 3:**
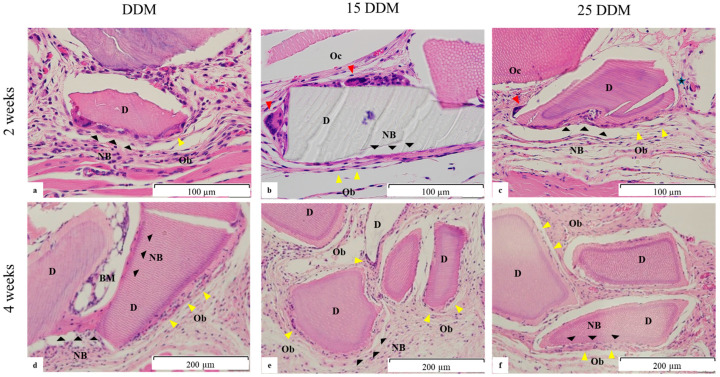
Representative histological images of the DDM, 15DDM, and 25DDM in the subcutaneous pocket of the nude mice at two (**a**–**c**) and four weeks (**d**–**f**). (**a**) DDM, new deposits of osteoid (NB, black arrowhead) were observed on the surface of the DDM (D) that were lined by prevalent osteoblasts (Ob, yellow arrowhead). (**b**) 15DDM, new deposits of osteoid (NB, black arrowhead) were observed on the surface of the DDM particles that were mainly lined by osteoblasts and some osteoclast-like cells (Oc, red arrowhead). (**c**) 25DDM, new deposits of osteoid (NB) were observed on the surface of the DDM. These were mainly lined by osteoblasts (Ob) and a few osteoclast-like cells (Oc). (**d**). DDM at four weeks, new bone bridges (NB) are formed between the DDM particles with osteocytic embeddings. Bone marrow (BM)-like structure is developed. (**e**,**f**) 15DDM and 25DDM at four weeks, a few microns of newly deposited osteoid (NB) are observed on the surface of the DDM particles. These were mainly lined by osteoblasts (Ob). NB; new bone, D; DDM; demineralized dentin matrix, Ob; osteoblast, Oc; osteoclast-like cell, BM; bone marrow.

Osteoinduction associated with DDM grafts could be observed in decalcified sections, as shown in Figure 3a. Non-irradiated DDM induces new bone formation around the DDM fragments. New bone bridges were formed between DDM particles, and bone marrow-like structures were developed at 4 weeks (Figure 3d). In contrast, 15DDM and 25DDM were surrounded by dense connective tissue, with a few microns of newly formed bone on the surface of the DDM (Figure 3b,c,e,f).

### 3.2. ALP and TRAP Staining at 4 Weeks

**Figure 4:**
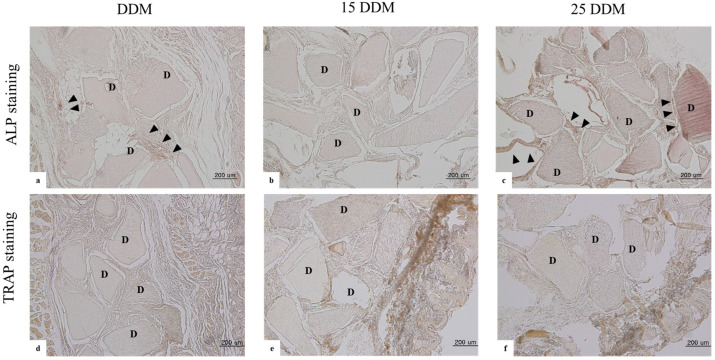
Immunohistochemical staining of DDM, 15DDM, and 25DDM at100× objective magnification. ALP staining shows positive activity in DDM control, and 25DDM shows alkaline phosphatase at four weeks (arrowhead in (**a**,**c**)). However, there was no positive activity of ALP staining in 15DDM (**b**). TRAP staining shows negative activity at four weeks in all control and experimental groups (**d**–**f**). ALP stained the active osteoblasts at the newly formed bone area, while TRAP stained osteoclasts around the resorbed dentin particle. D; DDM; demineralized dentin matrix, TRAP; tartrate-resistant acid phosphatase, ALP; alkaline phosphatase.

Alkaline phosphatase (ALP) is the key enzyme for osteoblast differentiation and bone calcification in osteoblasts. Considering the control samples, positive ALP staining is reddish-brown, while positive TRAP staining is purple (Supplementary Figure S3). DDM and 25DDM showed positive activity compared to 15DDM at 4 weeks (Figure 4a–c). In tartrate-resistant acid phosphatase (TRAP) staining to identify osteoclasts, all samples of DDMs showed negative osteoclastic activity regardless of gamma radiation dose (Figure 4d–f).

These in vivo ALP activities are well-correlated with the above histological findings, determining that representative ALP staining did not show any bone formation activity when exposed to 15 kGy radiation dosage (Figure 4b), while at a 25-kGy dosage, the osteoinductive activity was very similar to that of non-irradiated DDM (Figure 4a,c).

### 3.3. Number of Osteoblasts and Osteoclast-Like Cells on the Surface of the DDM

At two weeks, the number of osteoblasts was 5.3 ± 2.1, 5.7 ± 5.5, and 8.1 ± 3.9 in DDM, 15DDM, and 25DDM, respectively. At four weeks, the number of osteoblasts was 7.4 ± 5.4, 6.3 ± 2.5, and 6.9 ± 3.2 in DDM, 15DDM, and 25DDM, respectively. The numbers of osteoblasts are in line with the amount of new bone formation in each group and time point (Table 1). The number of osteoclast-like cells, not osteoclasts due to negative TRAP staining in all samples, was 7.2 ± 3.3, 6.6 ± 5.4, and 5.5 ± 3.7 in DDM, 15DDM, and 25DDM, respectively, at two weeks. At four weeks, the number of osteoclast-like cells was 8.5 ± 6.9, 3.7 ± 4.3, and 10.2 ± 6.67 in DDM, 15DDM, and 25DDM, respectively. There were no significant differences in the osteoblast and osteoclast-like cell numbers among the groups at each time point (Table 1).

### 3.4. Measurement of New Bone Formation

The amount of new bone formation was 17.8 ± 13.4, 16.8 ± 5.47, and 20.8 ± 10.4% in DDM, 15DDM, and 25DDM, respectively, at two weeks. At four weeks, the amount of new bone formation was 26.1 ± 22.4, 18.0 ± 9.3, and 23.0 ± 7.2% in DDM, 15DDM, and 25DDM, respectively. There was no statistical significance in new bone induction among the groups (Table 1).

### 3.5. Radiographic Evaluation of the Bone Mineral Density (g/cm^3^)

The BMD was 0.109, 0.095, and 0.092 g/cm^3^ in DDM, 15DDM, and 25DDM, respectively, at two weeks. At four weeks, the BMD was 0.128, 0.102, and 0.095 g/cm^3^ in DDM, 15DDM, and 25DDM, respectively. However, there were no statistically significant differences between all groups (Table 2).

## 4. Discussion

The objective of this study was to assess the effects of GR at doses of 15 and 25 kGy on the osteoinductivity of the DDM that had been demineralized and lyophilized. The authors hypothesized that the GR at both doses would not significantly affect the osteoinductivity of the non-irradiated DDM. Our study suggested that DDM showed osteoinductivity after 15 and 25 kGy GR treatment in nude mice. In particular, the amount of decreased new bone formation in the 25DDM was slightly less than that of the 15DDM, and it was similar to that of the non-irradiated DDM group at four weeks. However, the difference between the 15DDM and 25DDM groups did not reach statistical significance (Table 1).

This observation was not consistent with the results from in vitro DBM study that showed that active growth factors in the DBM are vulnerable to GR. Because DBM showed decreased bone formation when exposed to incremental radiation dosages in the histological findings, the new bone formation by DBM was inhibited essentially and completely after treatment with GR at a 25 kGy dosage [35]. On the other hand, this unusual phenomenon was also reported by Wientroub and Reddi [36], who observed that bone formation potential in DBM was increased instead of decreased after high-dose irradiation from 0 to 25 kGy. They demonstrated that denatured collagen by GR will more readily release growth factors in the DBM and shorten the osteoinductivity time. Therefore, it may not alter the collagen characteristic as a scaffold and a growth factor carrier. In the freeze-dried state, DBM could better withstand significant levels of GR while losing minimal osteoinductivity [35].

There were no chondrocytes observed around DDM, and the osteoblastic activity showed the intramembranous bone formation of DDM. Bone marrow-like structure was observed around DDM, while fibrous tissues were found around 15DDM and 25DDM. The numbers of ALP-positive osteoblasts were in line with the amount of new bone formation (Table 1). In addition, the multinucleated cells could not be confirmed as osteoclasts due to negative TRAP staining. Although multinucleated cells were shown on DDM regardless of GR at 2 and 4 weeks (Figure 1 and Figure 3), TRAP staining revealed that there were no osteoclasts on DDM, 15DDM, and 25DDM (Figure 4). Osteoblasts and osteoclasts, together with blood supply and associated connective tissue, assemble in the basic multicellular unit (BMU) with little morphological differences. During the bone matrix resorption by osteoclasts, the release of different factors, such as BMPs, induces osteoblasts to deposit new bone. Briefly, the osteoclastic resorption and osteoblastic bone matrix formation are part of a complex process identified as “coupling” [37]. Because the osteoclastic resorption was well-demonstrated for dentin (11-fold higher than that of bone [38]), the histology in this study showed the resorption of DDM by the multinucleated cells which is the unique function of the osteoclast [39]. Therefore, we regarded these multinucleated cells as osteoclast-like cells.

The numbers of osteoclast-like cells in the 15DDM and 25DDM groups were less than that in the non-irradiated DDM group at two weeks, although it showed a reverse tendency at four weeks. In contrast, in the human DBM, the number of TRAP-positive cells was significantly higher in the GR group at doses of 11, 15, and 22.5 kGy in comparison to the non-irradiated group [40,41]. It has been shown that the appearance of the osteoblasts precedes osteoclast infiltration during DBM-induced osteogenesis [42]. This is consistent with the ability of the BMPs within the DBM to induce the differentiation of the mesenchymal stem cells into osteoblasts, which then have the potential to regulate the production of osteoclasts. Regarding the DDM for the osteoblastic and osteoclastic results, these mixed results are probably a combination of the osteoclast-like cell and osteoblast interactions along with the other unknown interactions.

The BMD was showed no statistically significant differences between the groups (Table 2). However, in a study of human DBM treated with 11 kGy GR, a calcium deposition at six weeks showed denser images for the non-irradiated samples in rat intramuscular pockets [40]. The mineral density associated with the DBM appeared to decrease with an increase in the GR dosage from 0 to 25 kGy [35]. Considering the effects of GR on DBM, it was postulated that the collagen scaffold became more readily biodegraded by GR [36]. In 2008, an in vivo study showed the osteoinductivity of the DBM appeared to decrease with an increase in the GR dose when it was set to 25 kGy during the intramuscular implantation of athymic nude rats [35]. In 2014, Wong et al. reported that the mean new bone formation was higher in the non-irradiated DBM (21.4%) than in the gamma-irradiated DBM (15.3%), although the difference was not statistically significant [40]. Given that GR generally decreases the DBM osteoinductivity in a dose-dependent manner [43,44], GR with 10 to 35 kGy is considered to be an accepted method in a survey of tissue banks [45]. Similarly, the osteoinductivity of DDM was not significantly affected by GR doses between 0 and 25 kGy at each time point (Table 1 and Table 2).

DBM collagen molecules are susceptible to damage by GR at dosages commonly used for sterilizing biomedical products (0–15 kGy) [46]. The biological damage could be direct damage that results in the breakage of covalent bonds of collagen fibers, or indirect damage that is responsible for protein denaturation [35]. Even though the covalent bonds of collagen are cleaved by GR, GR may also introduce extra inter- or intra-molecular cross-links into collagen to stabilize the fibril structure [47], which may contribute to maintaining the properties of the scaffold and the growth factor carrier [48].

GR may inactivate some growth factors in DBM [35], but BMPs may appear to be less labile than collagen if it is irradiated separately [46]. In 2015, Kayal et al. demonstrated that GR with 25 kGy reduced the BMP-2 and BMP-7 percentage in DBM to 22% and 21%, respectively, in comparison to the non-irradiated DBM [49]. The DDMs retain BMP activity in the insoluble organic matrix (98% collagen) after removing the soluble components since the collagen fibril may be the locus of the BMPs [50]. In addition, the bone morphogenetic pattern was more stable in the dentin than in the bone matrix because of the highly cross-linked structure of the fibrous (insoluble) protein of the dentin, high density, and small surface area [51,52,53]. Taken together, DDM and DBM are collagenous materials that are impregnated with active growth factors. Since DDM does not have vascular channels nor a marrow space [51], the negative effect of irradiation could be diminished on DDM in comparison to DBM. In addition, the low antigenicity of DDM, due to the acellular nature of dentin, could reduce the irradiation dose required to reduce the risk of viral disease transmission [3,22,54,55].

Because this is the first report on the effect of GR on the osteoinductive properties of DDM, it is only possible to extrapolate the results of this study on the basis of the previous studies on the DBM. Taking into consideration the limitations of this study, DDM with GR might be effectively compared to DBM in terms of an allogeneic osteoinductive bone substitute. However, the exact mechanism of the action of GR on DDM collagen and its growth factors are unknown. The relationship between the most appropriate GR dose for viral clearance and the biological impact on the osteoinductivity of DDM should be further investigated.

## 5. Conclusions

Regardless of gamma radiation at 15 and 25 kGy, the activity of osteoclast-like cells and osteoblast is confirmed on the DDM, and new bone is induced in the extra-skeletal site. Therefore, this study suggests that gamma radiation at a 15 and 25 kGy dosage does not affect the osteoinduction capacity of DDM. However, the effects of gamma radiation on DDM have to be carefully monitored if it is ever to be used as a method for sterilizing DDM prior to implantation or as part of a bioactive composite because of its potential to inactivate osteoinductive factors.

## Figures and Tables

**Table 1:** Number of osteoblasts and osteoclast-like cells and the areas of new bone formation on the surface of the DDM.

	At Two Weeks			*p* *	At Four Weeks			*p* *
	DDM (*n* = 10)	15DDM (*n* = 5)	25DDM (*n* = 5)		DDM (*n* = 10)	15DDM (*n* = 5)	25DDM (*n* = 5)	
Osteoblast (N)	5.3 ± 2.1	5.7 ± 5.5	8.1 ± 3.9	0.271	7.4 ± 5.4	6.3 ± 2.5	6.9 ± 3.2	0.821
Osteoclast-like cells (N)	7.2 ± 3.3	6.6 ± 5.4	5.5 ± 3.7	0.666	8.5 ± 6.9	3.7 ± 4.3	10.2 ± 6.6	0.064
New bone (%)	17.8 ± 13.4	16.8 ± 5.47	20.8 ± 10.4	0.840	26.1 ± 22.4	18.0 ± 9.3	23.0 ± 7.2	0.697

Data were expressed as a mean ± standard deviation. * One-way ANOVA among the DDM, 15DDM, and 25DDM groups.

**Table 2:** Measurements of the bone mineral density by dual-energy X-ray absorptiometry.

	Bone Mineral Density (g/cm^3^)			*p* *
	DDM	15DDM	25DDM	
2 weeks	0.109	0.095	0.092	>0.999
4 weeks	0.128	0.102	0.095	>0.999

* One-way ANOVA among the DDM, 15DDM, and 25DDM groups.

## Data Availability

Not applicable

## Supplementary Materials

The following supporting information can be downloaded at: https://www.mdpi.com/article/10.3390/jfb13010014/s1, Figure S1: A split-designed experiment was performed on both thighs. One was grafted DDM without GR treatement as control group, and the other was grafted DDM with GR treatment at 15 or 25 kGy as experimental group.; Figure S2: The cell counting boundary. The boundary was marked and then at 25 μm distance around the perimeter of the DDM particle. The cell counting was done in this area.; Figure S3: The positive and negative control of ALP and TRAP staining. Positive ALP staining is reddish brown, while positive TRAP staining is purple.

## Abbreviations

DDM	Non-irradiated demineralized dentin matrix
15DDM	Gamma irradiated demineralized dentin matrix with a 15 kGy dosage
25DDM	Gamma irradiated demineralized dentin matrix with a 25 kGy dosage
GR	Gamma irradiation
