# Supplementary material for: Effect of Gamma Irradiation on the Osteoinductivity of Demineralized Dentin Matrix for Allografts: A Preliminary Study
Source: J Funct Biomater. 2022 Jan 31;13(1):14. doi: 10.3390/jfb13010014 (PMC8883982; doi:10.3390/jfb13010014)
Supplement: Supplementary file 1 [file jfb-13-00014-s001.zip › jfb-1565342-supplementary.pdf]

Journal of
Functional Biomaterials

Supplementary

Effect of Gamma Irradiation on the Osteoinductivity of a Demineralized Dentin Matrix for Allografts: A Preliminary Study

Jeong-Kui Ku [1], Il-hyung Kim [2], In-Woong Um [3,*], Bo-Hyun Kim [3] and Pil-Young Yun [4,*]

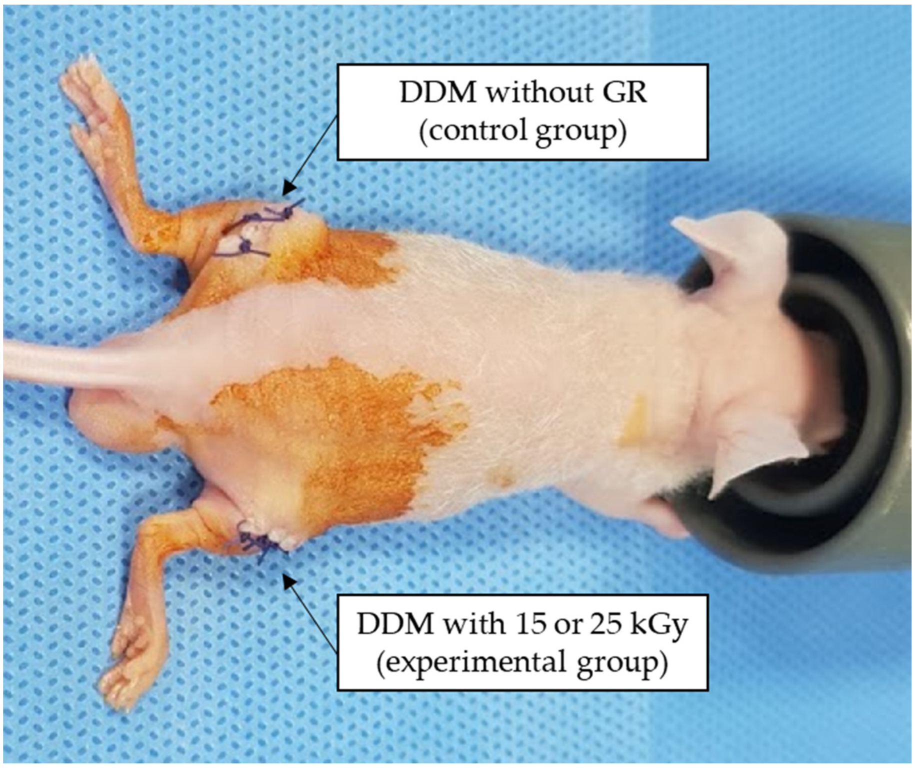

Figure S1. Experimental design**.** A split-designed experiment was performed on both thighs. One was grafted DDM without GR treatement as control group, and the other was grafted DDM with GR treatment at 15 or 25 kGy as experimental group.

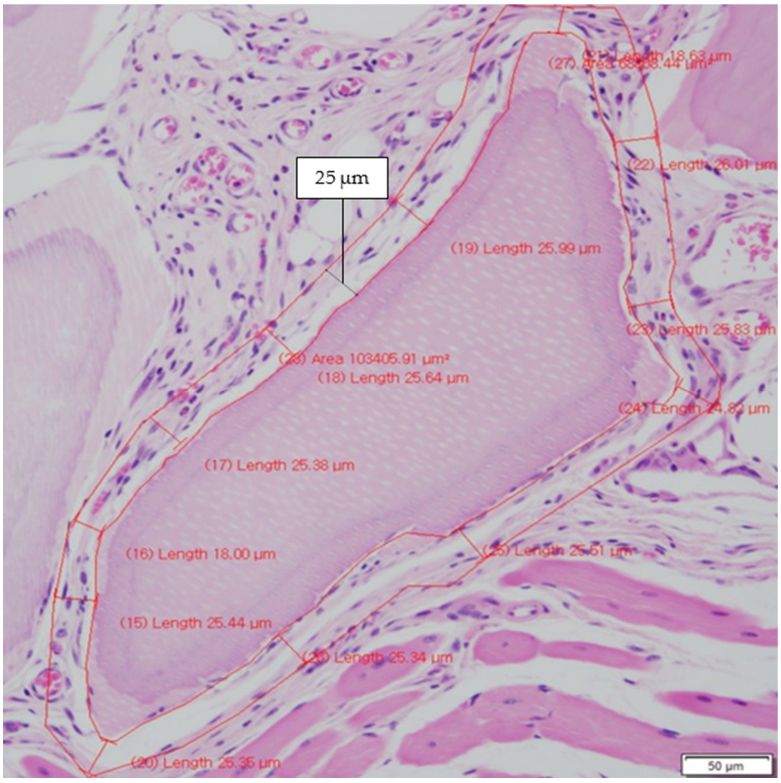

Figure S2. The cell counting boundary. The boundary was marked and then at 25 μm distance around the perimeter of the DDM particle. The cell counting was done in this area.

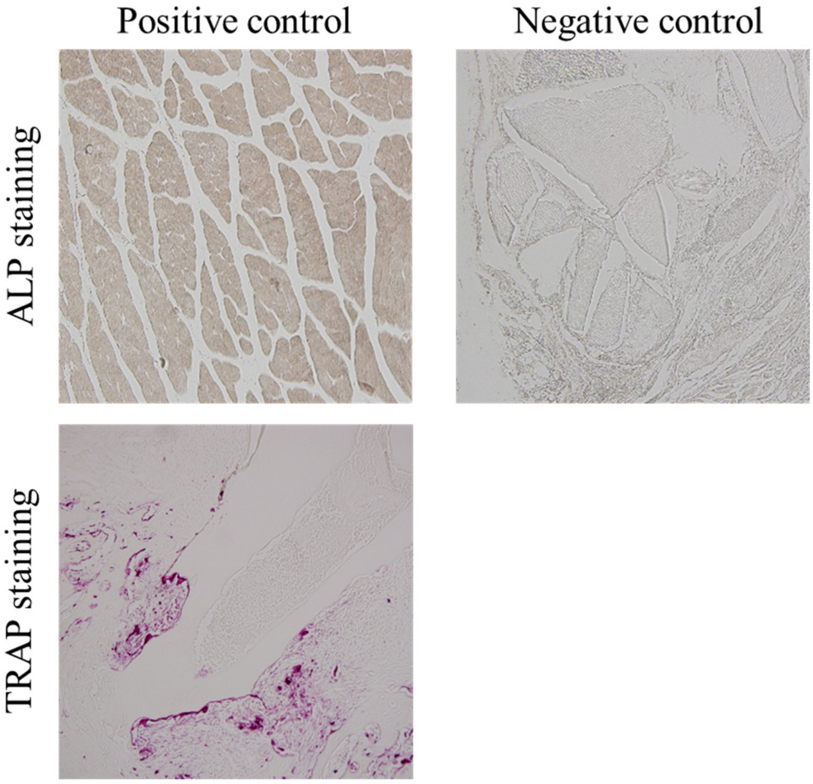

Figure S3. The positive and negative control of ALP and TRAP staining. Positive ALP staining is reddish brown, while positive TRAP staining is purple.
